# Phosphodiesterase type 5 inhibitors combined with traditional Chinese medicine for diabetes mellitus-induced erectile dysfunction: A systematic review and meta-analysis

**DOI:** 10.1097/MD.0000000000043243

**Published:** 2025-07-11

**Authors:** Lei Xiang, Aiqun Liu, Wenyi Tang, Bin Sun, Yang Yang, Yilin Li, Siyu Chen, Juyu Zhao, Weizheng Zhang, Xiangsheng Cai, Xiangjiang Tang

**Affiliations:** a Department of Traditional Chinese Medicine, Guangzhou Cadre and Talent Health Management Center, Guangzhou 11th People’s Hospital, Guangzhou, China; b Department of Neurology Internal Medicine, The First Affiliated Hospital of Guangdong Pharmaceutical University, Guangzhou, China; c Clinical Laboratory, Guangzhou Cadre and Talent Health Management Center, Guangzhou 11th People’s Hospital, Guangzhou, China.

**Keywords:** diabetes mellitus-induced erectile dysfunction, meta-analysis, phosphodiesterase type 5 inhibitors, traditional Chinese medicine

## Abstract

**Background:**

Diabetes mellitus-induced erectile dysfunction (DMED) involves complex pathophysiology, leading to suboptimal outcomes with phosphodiesterase type 5 inhibitors (PDE5I) alone. Combining PDE5I with traditional Chinese medicine (TCM) may enhance therapeutic efficacy, but evidence synthesis is lacking. Therefore, this systematic review and meta-analysis aims to compare the efficacy and safety of PDE5I combined with TCM versus PDE5I monotherapy in DMED management.

**Methods:**

We conducted a systematic review and meta-analysis following Preferred Reporting Items for Systematic Reviews and Meta-Analyses 2020 guidelines (PROSPERO: CRD42021240608). Six databases (PubMed, Cochrane Library, Web of Science, China National Knowledge Infrastructure Database, Wanfang, and VIP Science Technology Periodical Database) were searched up to May 10, 2024. Randomized controlled trials (RCTs) evaluating PDE5I + TCM versus PDE5I alone in DMED patients with International Index of Erectile Function-5 (IIEF-5) ≤ 21 were included. Data extraction, risk of bias assessment (Cochrane tool), and meta-analysis (RevMan 5.3) were performed using fixed/random-effects models. Outcomes included clinical efficacy, IIEF-5 scores, TCM syndrome scores, and adverse events.

**Results:**

Twelve RCTs (1070 participants) were included. PDE5I + TCM significantly improved clinical efficacy (relative risk = 2.86, 95% confidence interval [CI] [2.13, 3.84], *P* < .001) compared to PDE5I alone. Subgroup analysis showed higher IIEF-5 scores for tadalafil + TCM (standardized mean difference [SMD] = 1.07, 95% CI [0.85, 1.30], *P* < .001) and sildenafil + TCM (SMD = 1.38, 95% CI [1.11, 1.65], *P* < .001). TCM syndrome scores decreased significantly with combination therapy (tadalafil + TCM: SMD = -3.43, 95% CI [-3.93, -2.94]; sildenafil + TCM: SMD = -1.40, 95% CI [-1.77, -1.03]). Adverse event rates (dizziness, gastrointestinal effects, flushing) did not differ between groups (all *P* > .05).

**Conclusion:**

This study demonstrates that PDE5I combined with TCM demonstrates superior efficacy in improving erectile function and TCM symptom profiles in DMED patients, without increasing adverse events. However, limitations such as variability in TCM formulations and treatment durations across studies, as well as unclear blinding protocols in some trials, may introduce heterogeneity and affect the generalizability of these findings. Further high-quality RCTs are needed to validate optimal regimens and long-term outcomes.

## 1. Introduction

Diabetes mellitus-induced erectile dysfunction (DMED) is a type of erectile dysfunction observed in people with diabetes.^[1]^ Erectile dysfunction (ED), known as “impotence,” is the inability to achieve or maintain an erection strong enough for satisfactory sexual performance.^[2,3]^ A meta-analysis of 145 studies revealed that the prevalence of ED in diabetes was 52.5%.^[4]^ The oral phosphodiesterase type 5 inhibitors (PDE5I), a first-line therapy, with the highest recommended levels for treating DMED. However, DMED exhibits pathological features of diabetes and erectile problems, making it challenging to manage DMED with only PDE5I. Patients with ED and diabetes present apparent resistance against PDE5I treatment.^[5]^ The effective rate of syndrome relief in patients with ED and diabetes was significantly lower than that in patients with ED who did not have diabetes.^[6]^ For instance, sildenafil treatment, the total remission rate of patients with ED who did not have diabetes was approximately 83%,^[7]^ whereas only 52% of patients with DMED treated with sildenafil reported improved erections.^[8]^ Therefore, it is needed for a new and reliable treatment method to help improve the therapeutic effect of DMED.

In recent years, traditional Chinese medicine (TCM) combined with PDE5I has enhanced ED efficacy in real-world investigations, randomized controlled trials (RCTs). Sildenafil combined with TCM can elevate the International Index of Erectile Function-5 (IIEF-5) score of ED among Chinese men.^[9]^ Combining Shanhaidan Granules and tadalafil can promote erectile function and improve TCM syndrome scores in patients with ED.^[10]^ A systematic review and meta-analysis demonstrated that tadalafil combined with TCM has definite efficacy in treating ED.^[11]^

Given the dual pathogenesis of DMED involving both metabolic dysregulation and vasculogenic impairment, there was a lack of a comprehensive systematic review of whether integrating PDE5I with TCM formulations can improve therapeutic efficacy without increasing side effects. This systematic review and meta-analysis aimed to quantitatively synthesize evidence from RCTs evaluating the risk-benefit profile of PDE5I and TCM combination therapy in the management of DMED, with specific focus on therapeutic efficacy, IIEF-5 score improvement, TCM syndrome scores and treatment-emergent adverse event incidence.

## 2. Materials and methods

### 2.1. Study protocol and registration

This systematic review and meta-analysis were performed based on the Guidelines of Systematic Reporting of Examination presented in the Preferred Reporting Items for Systematic Reviews and Meta-Analyses (PRISMA) criteria and the completed checklist is provided in Table S1, Supplemental Digital Content, https://links.lww.com/MD/P418.^[12]^ The study’s methodology was registered in the PROSPERO International Prospective Register of Systematic Reviews (No. CRD42021240608).

### 2.2. Inclusion criteria

The inclusion criteria of this study are as follows: (1) randomized controlled clinical trials. (2) The cases of DMED must fulfill the diagnostic criteria of diabetes and the score of IIEF-5 not more than 21 points. (3) Patients of DMED in the experimental group received PDE5I combined with TCM, whereas patients in the control group only received PDE5I. (4) The efficacy index must have an IIEF-5 score, which is specific and sensitive for assessing treatment-related changes in patients with ED.^[13]^

### 2.3. Exclusion criteria

The exclusion criteria of this study are as follows: (1) random grouping or random method is not clear or incorrect. (2) There is an interference of other drugs for ED in the test or control group. (3) The trial data did not provide any treatment effect or failed to achieve an IIEF-5 score. (4) There is a case of repeated literature, meta-analysis, or review literature.

### 2.4. Retrieval strategies

An exhaustive literature search was conducted using the following databases: PubMed, Cochrane Library, Web of Science, China National Knowledge Infrastructure Database, Wanfang Database, and VIP Science Technology Periodical Database. Related data in the included articles was extracted independently by 3 researchers (Aiqun Liu, Wenyi Tang, and Bin Sun) according to the PRISMA statement, and disputes were solved through adjudication and discussion by the other 3 reviewers (Siyu Chen, Xiangjiang Tang, and Xiangsheng Cai). All articles up to May 10, 2024, were retrieved for this study. The search did not exclude articles based on language. The following keywords and full search strategy for different databases were provided in Table S1, Supplemental Digital Content, https://links.lww.com/MD/P418.

### 2.5. Literature quality evaluation

The literature quality evaluation methods recommended by the Cochrane Risk of Bias Assessment tool, such as random sequence generation, allocation concealment, blinding of participants and personnel, blinding of outcome assessment, incomplete outcome data, selective reporting, and other biases, were applied in this study. Graph and summary about the risk of bias were produced with RevMan 5.3.

### 2.6. Statistical methods

Statistical analyses were conducted using RevMan 5.3 and displayed as a forest plot, while a funnel plot has been generated to assess the risk of bias. The heterogeneity test was performed before the meta-analysis. If there were no heterogeneity between the 2 groups (the *P*-value of the Q test is > .1 and I^2^ < 50%), the fixed-effect model was adopted. When significant heterogeneity was identified (the *P*-value of the Q test < .1 and I^2^ ≥ 50%), subgroup analyses were initiated, with subsequent adoption of a random-effects model if heterogeneity thresholds remained unmet. Sensitivity analysis was conducted to assess the robustness of the results. When there were more than 10 studies with combined effect amount, publication bias was analyzed using a funnel plot. Relative risk (RR) was used for counting data, standardized mean difference (SMD) was selected for measurement data, and a 95% confidence interval (95% CI) was applied as a combined effect amount. *P* values <.05 indicate statistically significant differences.

## 3. Results

### 3.1. Results of literature retrieval

**Figure 1.**
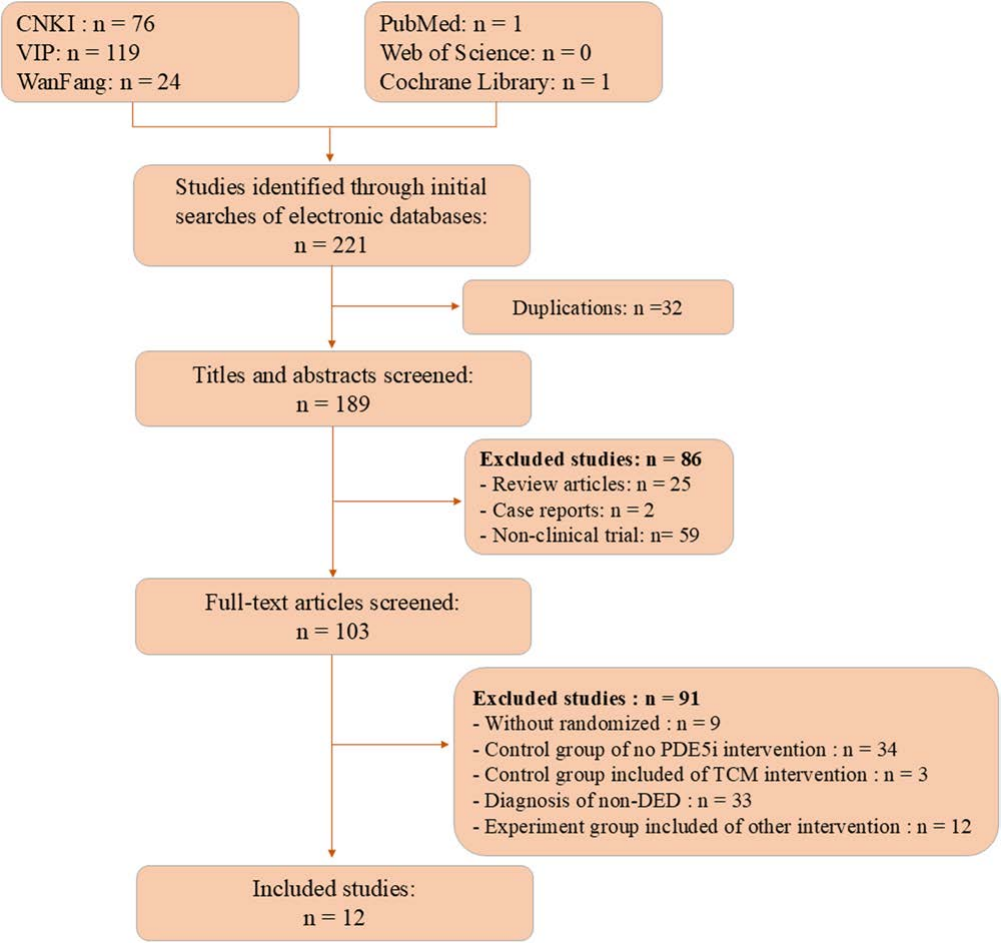
Flowchart of study selection.

A total of 221 reports were retrieved from 6 databases. Among them, 32 papers were eliminated because of duplication, and 86 was removed by reading their titles and abstracts. Of the remaining 103 reports, 91 did not fulfill the inclusion requirements and were excluded after their full texts were read. Finally, 12 eligible papers^[14–25]^ were included in this study (Fig. 1). In total, 540 cases were included in the experimental group, aged between 18 and 72 years, with the course of DMED ranging from 2 months to 14 years. The control group had 530 cases, aged between 18 and 78 years, with a duration of DMED ranging from 2 months to 15 years (Table 1).

**Table 1:** Characteristics of included studies.

First author	Publication year	E/C No. of cases	Intervention (drug and dose)		Treatment duration (wk)
			Experimental group	Control group	
Li XR	2022	41/41	Tianjing Tongluo Decoction, 200 mL, bid. Tadalafil, 5 mg, qd	Tadalafil, 5 mg, qd	4
Sun S	2021	33/33	Huoxue Tongluo Qiwei Decoction, 200 mL, bid. Tadalafil, 5 mg, qd	Tadalafil, 5 mg, qd	4
Jia XY	2021	41/41	Shaofu Zhuyu Decoction, 200 mL, bid. Sildenafil 50 mg, qd	Sildenafil 50 mg, qd	8
Wu QH	2023	39/39	Yishen Tongluo Decoction, 200 mL, bid. Tadalafil, 5 mg, qd	Tadalafil, 5 mg, qd	8
Yang ZG	2019	30/30	Xionglou Tongmai Decoction, about 200 mL, bid. Sildenafil, 25 mg, qd	Sildenafil, 25 mg, qd	8
Yang H	2017	60/60	Shianwei Ziyin Zhuangyang Capsule, 3 capsules, tid. Sildenafil, 25 mg, qd	Sildenafil, 25 mg, qd	4
Liu LH	2017	90/90	Wenshen Huoxue Tongluo Decoction, 200 mL, qd. vardenafil, 5–20 mg, qd	Vardenafil, 5–20 mg, qd	12
Zhao F	2020	45/45	Qiyang capsule, 2.5 g, tid. Tadalafil, 5 mg, qd	Tadalafil 5 mg, qd	4
Zhou H	2015	34/32	Erdi Biejia Decoction, 200 mL, bid. Tadalafil, 5 mg, qd	Tadalafil, 5 mg, qd	8
Wang JZ	2019	31/31	Xuanju capsule, 1.26 g, tid. Tadalafil 5 mg qd	Tadalafil, 10 mg, qd	4
Luo ES	2019	32/32	Xuefu Zhuyu tablet, 6 tablets, tid. Tadalafil, 5 mg, qd	Tadalafil 5 mg, qd	12
Wang XK	2009	64/60	Liuwei Dihuang Decoction, 200 mL, bid. Sildenafil, 50 mg, qd	Sildenafil, 50 mg, qd	12

E/C = experimental group/control group, IIEF-5 = International Index for Erectile Function-5, TCM = traditional Chinese medicine.

### 3.2. Results of risk of bias and literature quality

**Figure 2.**
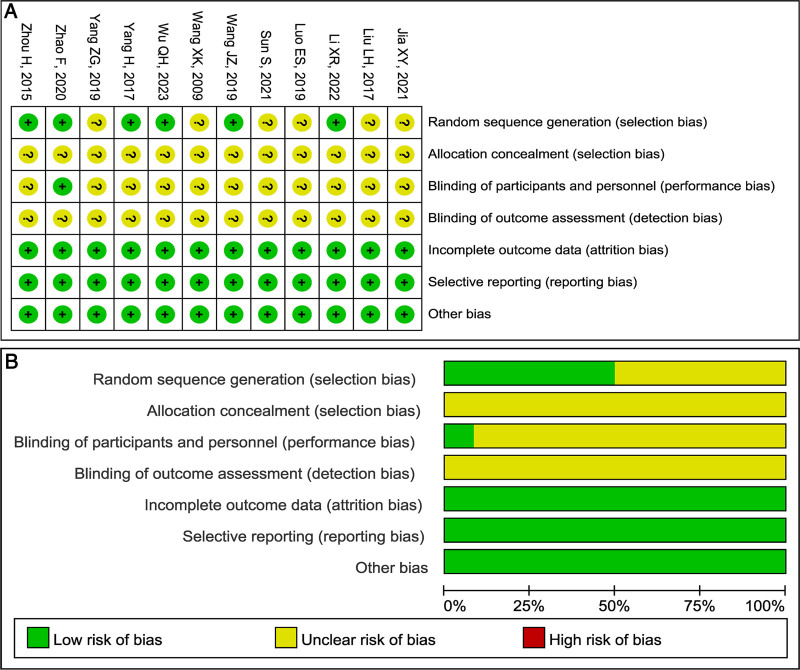
Risk of bias graph. (A) Review each risk of bias item for each included study. (B) Each risk of bias item presented as percentages across all included studies.

All included RCTs exhibited a low risk of attrition, reporting biases based on the Cochrane Collaboration’s tool for assessing the risk of bias. Full studies presented an unclear risk of detection biases and allocation concealment selection bias. Eleven showed an unclear risk of performance bias.^[14–20,22–25]^ Six studies^[15,17,19,21,24,25]^ depicted a low risk of random sequence generation selection bias (Fig. 2).

### 3.3. Therapeutic efficiencies

**Figure 3.**
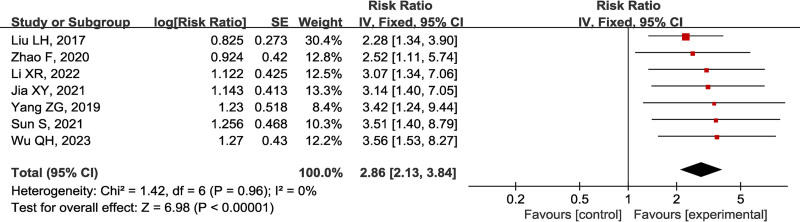
Forest plot of therapeutic efficiency between PDE5I alone and combination therapy groups for DMED. DMED = diabetes mellitus-induced erectile dysfunction, PDE5I = phosphodiesterase type 5 inhibitors.

Seven studies^[16,20–25]^ provided therapeutic efficiency data. The cumulative ratio model was applied for analysis. First, the ordered logistic regression analysis was conducted to calculate the effect quantity and its standard error. Second, the Generic Inverse Variance of RevMan 5.3 was used for consolidating effect quantity. The heterogeneity analysis showed no significant heterogeneity in 7 studies (I² = 0%, *P* = .96). Therefore, the fixed-effect model was adopted. The results revealed that the effective clinical rate of the experimental group was higher than that of the control group, and the difference was statistically significant [RR = 2.86, 95% CI (2.13, 3.84), *P* < .001]. The sensitivity analysis discovered that eliminating each study one by one had no essential change to the effect quantity. The results showed that compared with PDE5I treatment alone, the combined treatment could improve the therapeutic efficiency by one or more levels was 2.86 times (Fig. 3).

### 3.4. IEEF-5 scores

**Figure 4.**
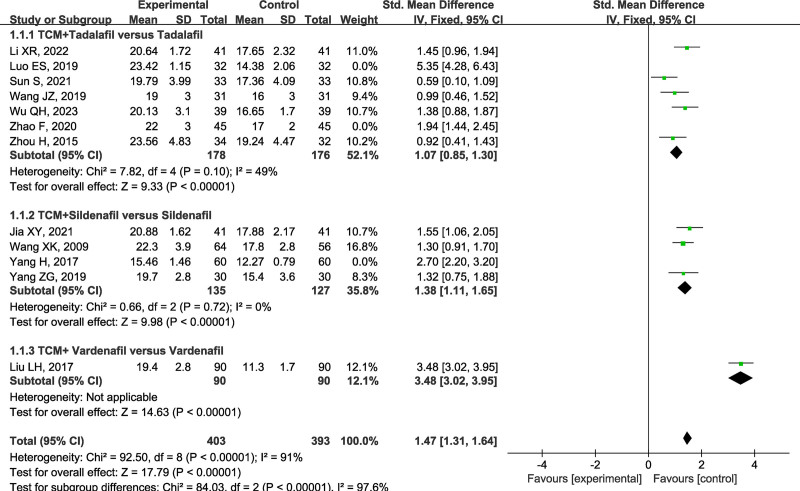
Forest plot of subgroup analysis for IIEF-5 scores based on different types of PDE5I. IIEF-5 = International Index for Erectile Function-5, PDE5I = phosphodiesterase type 5 inhibitors.

Twelve literatures achieved IEEF-5 scores between the control group and the treatment group. The higher the IEEF-5 scores, the more pronounced the subjective improvement of clinical syndrome. The heterogeneity test revealed high heterogeneity between the included studies (I² = 96%, *P* < .001). The subgroup analysis was introduced by dividing them into 3 subgroups, namely tadalafil plus TCM group, sildenafil plus TCM group, and vardenafil plus TCM group, to compare the different kinds of PDE5I (Fig. 4).

(1) The tadalafil plus TCM group had 7 studies.^[15,18,19,21,23–25]^ The heterogeneity analysis presented a statistical difference (I² = 92%, *P* < .001), and sensitivity analysis were performed. After removing 2 studies,^[18,21]^ no significant heterogeneity was observed within the tadalafil group (I² = 49%, *P* = .10). The remaining 5 studies underwent meta-analysis, and a fixed-effect model was selected [SMD = 1.07, 95% CI (0.85, 1.30), *P* < .001]. This outcome shows that tadalafil combined with TCM can significantly improve the IEEF-5 scores.

(2) The sildenafil plus TCM group had 4 studies.^[14,17,20,22]^ The heterogeneity analysis yielded significant heterogeneity (I² = 86%, *P* = .11), and sensitivity analysis were performed. After removing the study,^[17]^ no significant heterogeneity was observed within the sildenafil group (I² = 0, *P* = .72). The remaining 3 studies^[14,20,22]^ underwent meta-analysis, and a fixed-effect model was selected [SMD = 1.38, 95% CI (1.11, 1.65), *P < *.001]. It shows that sildenafil combined with TCM can significantly improve the IEEF-5 scores.

(3) The vardenafil plus TCM group had only 1 eligible paper,^[16]^ this study did not do a further combined effect size analysis of IIEF-5 scores.

### 3.5. TCM syndrome scores

**Figure 5.**
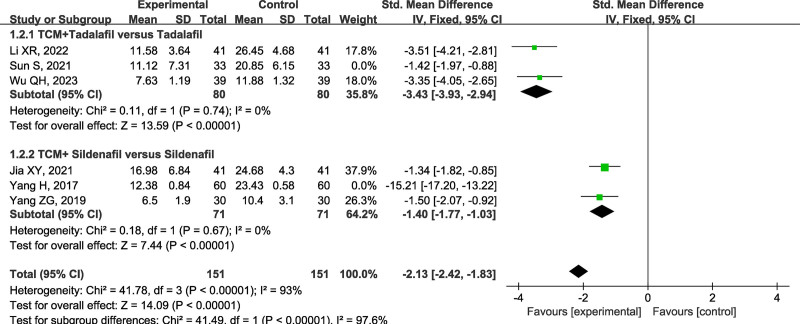
Forest plot of subgroup analysis for TCM syndrome scores between PDE5I alone and combination therapy groups for DMED. DMED = diabetes mellitus-induced erectile dysfunction, PDE5I = phosphodiesterase type 5 inhibitors, TCM = traditional Chinese medicine.

Six included studies^[17,20,22–25]^ employing standardized TCM syndrome differentiation protocols reported quantifiable TCM syndrome scores, with lower scores indicating superior therapeutic efficacy. However, significant between-study heterogeneity was detected (I² = 98%, *P* < .001), subsequent subgroup analyses to identify potential moderators. The subgroup analysis was performed by stratifying them into 2 subgroups, namely tadalafil plus TCM group, and sildenafil plus TCM group, according to the type of PDE5I administered (Fig. 5).

(1) The tadalafil plus TCM group had 3 studies.^[23–25]^ The heterogeneity analysis presented a statistical difference (I² = 93%, *P* < .001), and sensitivity analysis were performed. After removing 1 study,^[23]^ no significant heterogeneity was observed within the tadalafil group (I² = 0%, *P* = .74). The remaining 2 studies underwent meta-analysis, and a fixed-effect model was selected [SMD = -3.43, 95% CI (-3.93, -2.94), *P* < .001]. The results demonstrated significantly lower TCM syndrome scores in the tadalafil plus TCM compared to tadalafil monotherapy group.

(2) The sildenafil plus TCM group had 3 studies.^[17,20,22]^ The heterogeneity analysis yielded significant heterogeneity (I² = 99%, *P* < .001), and sensitivity analysis were performed. After removing the study,^[17]^ no significant heterogeneity was observed within the sildenafil group (I² = 0, *P* = .67). The remaining 2 studies underwent meta-analysis, and a fixed-effect model was selected [SMD = -1.40, 95% CI (-1.77, -1.03), *P < *.001]. The results demonstrated significantly lower TCM syndrome scores in the sildenafil plus TCM combination group compared to the sildenafil monotherapy group.

### 3.6. Adverse events evaluation

**Figure 6.**
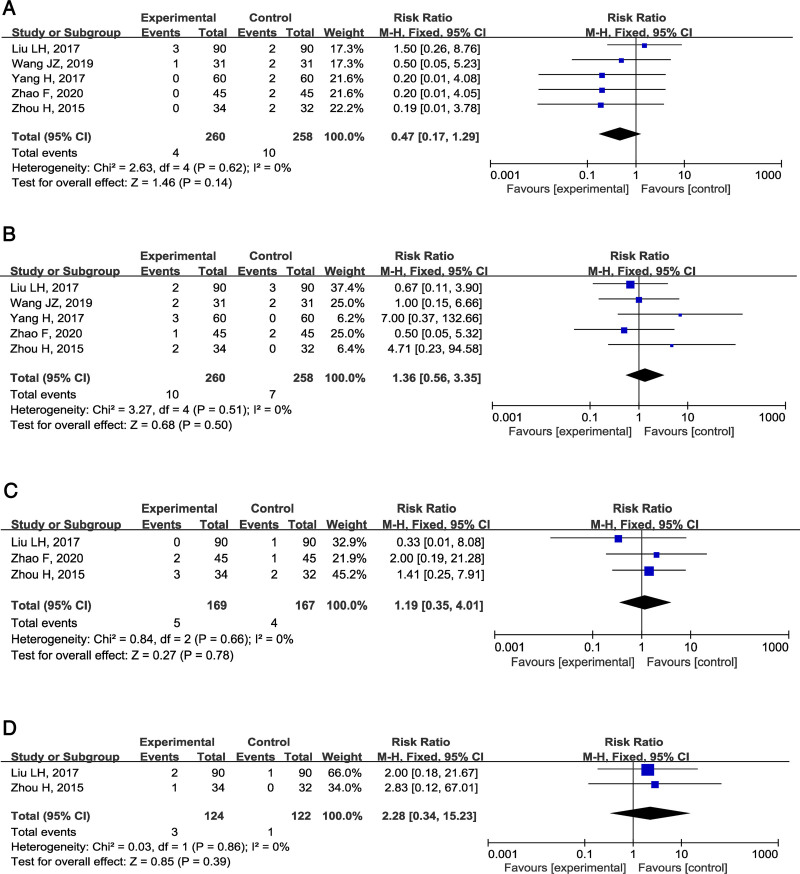
Forest plot of adverse events evaluation between PDE5I alone and combination therapy groups for DMED. (A) Dizziness and headache. (B) Dyspepsia, nausea, and diarrhea. (C) Flushing. (D) Sinusitis. DMED = diabetes mellitus-induced erectile dysfunction, PDE5I = phosphodiesterase type 5 inhibitors.

Five studies^[15–17,19,21]^ documented adverse events including dizziness, headache, dyspepsia, nausea, diarrhea, flushing, and sinusitis. (1) Meta-analysis of 5 studies demonstrated neurological treatment-emergent adverse events, specifically dizziness and headache, with no significant inter-study heterogeneity (I² = 0%, *P = *.62). The fixed-effect model analysis revealed no statistically significant differences between the experiment and control groups [RR = 0.47, 95% CI (0.17, 1.29), *P* = .14] (Fig. 6A). (2) Five studies documented gastrointestinal adverse events, including dyspepsia, nausea, and diarrhea, with no significant inter-study heterogeneity (I² = 0%, *P* = .51). The results from a fixed-effect model presented no statistical difference [RR = 1.36, 95% CI (0.56, 3.35), *P = *.50] (Fig. 6B). (3) Flushing was reported in 3 studies^[15,16,21]^ along with no significant heterogeneity (I² = 0%, *P* = .66). The fixed-effect model analysis revealed no statistically significant differences [RR = 1.19, 95% CI (0.35, 4.01), *P* = .78] (Fig. 6C). (4) Sinusitis was reported in 2 studies,^[15,16]^ with no significant heterogeneity (*I²* = 0%, *P* = .86). No statistically significant differences were observed in the fixed-effect model analysis [RR = 2.28, 95% CI (0.34, 15.23), *P* = .39] (Fig. 6D). The meta-analysis revealed that concomitant administration of PDE5I and TCM was not associated with increased risk of treatment-emergent adverse events in patients with DMED compared to PDE5I monotherapy.

### 3.7. Publication bias

Funnel plots to assess publication bias were not constructed due to the inclusion of fewer than 10 studies reporting effect sizes.

## 4. Discussions

DMED, a multifactorial and highly prevalent sexual disorder in men, profoundly impairs quality of life. While PDE5I remain first-line therapy for ED, their efficacy in diabetic populations is notably attenuated compared to non-diabetic individuals.^[26]^ This diminished therapeutic response likely stems from the complex pathophysiology of DMED, which involves intertwined mechanisms such as chronic oxidative stress, endothelial dysfunction, neurogenic impairment, and microvascular abnormalities.^[27]^ Hyperglycemia-driven depletion of endogenous antioxidants and accumulation of reactive oxygen species exacerbate oxidative damage, triggering cellular apoptosis within penile tissues.^[28]^ Concurrently, endothelial dysfunction (characterized by impaired nitric oxide [NO] bioavailability) compromises vasodilation and vascular homeostasis, a hallmark of DMED pathogenesis.^[29]^ Diabetic neuropathy further disrupts autonomic regulation of cavernosal blood flow, while structural alterations in penile vasculature (e.g., atherosclerosis, microangiopathy) and plaque-induced occlusion of penile arteries collectively impede erectile hemodynamics.^[30–32]^ These multifactorial challenges underscore the need for multimodal therapeutic strategies beyond conventional PDE5I monotherapy.

Emerging evidence highlights the potential of TCM to address these limitations through complementary mechanisms. Preclinical studies demonstrate that bioactive TCM constituents target key pathways implicated in DMED. For instance, GouQiZi (*Lycium barbarum*) polysaccharides mitigate oxidative stress, reducing penile erection latency in semi-castrated rat models.^[33]^ Icariin (derived from *Epimedium brevicornum*, YinYangHuo) preserves penile hemodynamics and neuronal NO synthase expression via transforming growth factor beta 1/SMAD family member 2 pathway modulation in streptozotocin-induced diabetic rats.^[34]^ Chuanxiongzine (from *Ligusticum chuanxiong*, ChuanXiong) enhances intracavernous pressure and relaxes corpus cavernosum smooth muscle by inhibiting cyclic guanosine monophosphate/cyclic adenosine monophosphate-specific phosphodiesterases.^[35]^ Clinical and experimental data further support the efficacy of TCM formulations in DMED management. Huoxue Tongluo Qiwei Decoction attenuates vascular endothelial damage in diabetic rats by suppressing protein kinase Cβ pathway activation and enhancing antioxidant capacity.^[36]^ Xuanju Capsule reduces serum levels of advanced glycation end products and angiotensin II, ameliorating erectile function in diabetic patients.^[37]^ Preclinical and clinical studies collectively demonstrate that TCM addresses the therapeutic limitations of PDE5I in DMED through complementary mechanisms, offering synergistic efficacy in both functional and pathological improvements.^[38,39]^

This meta-analysis was conducted systematically in accordance with Cochrane and PRISMA guidelines. Using the cumulative ratio model to evaluate therapeutic efficacy, we found that the combination of PDE5I and TCM significantly enhanced clinical efficacy, yielding a 2.86-fold improvement compared to monotherapy. Subgroup analysis of IIEF-5 scores (a widely recognized international scale for assessing ED severity and treatment responsiveness) revealed that both tadalafil and sildenafil, when combined with TCM, produced statistically meaningful improvements. However, the limited number of studies involving vardenafil precluded pooled effect size analysis for this subgroup. Additionally, the meta-analysis demonstrated that combination therapy significantly reduced TCM syndrome scores, reflecting amelioration of syndrome-specific manifestations in accordance with TCM diagnostic criteria. Safety evaluations indicated that the most commonly reported adverse events (including dizziness, headache, dyspepsia, nausea, diarrhea, flushing, and sinusitis) were consistent with known PDE5I profiles, and the combination regimen did not significantly increase adverse event incidence compared to PDE5I monotherapy. These findings suggest that PDE5I-TCM combination therapy represents a promising therapeutic strategy for DMED.

Although this meta-analysis revealed that the combined treatment was well tolerated in patients and effectively improved the IIEF-5 scores, decreased TCM syndrome scores, the limitations of this study should be considered when interpreting these results. Firstly, due to the limited number of clinical studies on specific Chinese herbal formulas or single herbs, this research only conducted a meta-analysis at a macro level comparing the combined use of PDE5I with TCM versus PDE5I alone. It did not analyze the effects of any specific Chinese herbal formula or single herb. Secondly, the included study did not consider the differences in the treatment course of TCM, whose drug administration time ranges from 4 to 12 weeks. Therefore, a relatively definite course of treatment is needed to determine the efficacy of combined treatment. Finally, the accurate dosage in combination therapy for DMED is still required for long-term, multicenter, randomized, double-blind clinical trials.

## 5. Conclusion

PDE5I combined with TCM demonstrates superior efficacy in improving erectile function and TCM symptom profiles in DMED patients, without increasing adverse events. Further high-quality RCTs are needed to validate optimal regimens and long-term outcomes.

## Author contributions

**Conceptualization:** Xiangjiang Tang.

**Data curation:** Aiqun Liu, Wenyi Tang, Bin Sun, Yang Yang, Yilin Li, Siyu Chen, Juyu Zhao, Weizheng Zhang, Xiangsheng Cai.

**Formal analysis:** Yilin Li, Siyu Chen, Weizheng Zhang, Xiangsheng Cai.

**Funding acquisition:** Lei Xiang, Xiangsheng Cai, Xiangjiang Tang.

**Investigation:** Juyu Zhao, Weizheng Zhang, Xiangsheng Cai.

**Project administration:** Lei Xiang, Xiangsheng Cai.

**Writing – original draft:** Lei Xiang.

**Writing – review & editing:** Lei Xiang.

## Supplementary Material


