# Supplementary material for: Phosphodiesterase type 5 inhibitors combined with traditional Chinese medicine for diabetes mellitus-induced erectile dysfunction: A systematic review and meta-analysis
Source: Medicine (Baltimore). 2025 Jul 11;104(28):e43243. doi: 10.1097/MD.0000000000043243 (PMC12262951; doi:10.1097/MD.0000000000043243)
Supplement: Supplementary file 1 [file medi-104-e43243-s001.docx]

**Supplementary Materia**

**Table S1. Search strategies and the number of records according to different electronic database**

| Search strategy | Database | Num. of records |
| --- | --- | --- |
| (("diabetes mellitus"[MeSH Terms] OR ("diabetes"[All Fields] AND "mellitus"[All Fields]) OR "diabetes mellitus"[All Fields]) AND ("erectile dysfunction"[MeSH Terms] OR ("erectile"[All Fields] AND "dysfunction"[All Fields]) OR "erectile dysfunction"[All Fields]) AND ("medicine, chinese traditional"[MeSH Terms] OR ("medicine"[All Fields] AND "chinese"[All Fields] AND "traditional"[All Fields]) OR "chinese traditional medicine"[All Fields] OR ("traditional"[All Fields] AND "chinese"[All Fields] AND "medicine"[All Fields]) OR "traditional chinese medicine"[All Fields])) AND (clinical trial[Filter]) | PubMed | 1 |
| #1 Diabetes Mellitus, Type 2  #2 (Diabetes Mellitus, Noninsulin-Dependent):ab,ti,kw OR (Diabetes Mellitus, Ketosis-Resistant):ab,ti,kw OR (Diabetes Mellitus, Ketosis Resistant):ab,ti,kw OR (Ketosis-Resistant Diabetes Mellitus):ab,ti,kw OR (Diabetes Mellitus, Non Insulin Dependent):ab,ti,kw OR (Diabetes Mellitus, Non-Insulin-Dependent):ab,ti,kw OR (Non-Insulin-Dependent Diabetes Mellitus):ab,ti,kw OR (Diabetes Mellitus, Stable):ab,ti,kw OR (Stable Diabetes Mellitus):ab,ti,kw OR (Diabetes Mellitus, Type II):ab,ti,kw OR (NIDDM):ab,ti,kw OR (Diabetes Mellitus, Noninsulin Dependent):ab,ti,kw OR (Diabetes Mellitus, Maturity-Onset):ab,ti,kw OR (Diabetes Mellitus, Maturity Onset):ab,ti,kw OR (Maturity-Onset Diabetes Mellitus):ab,ti,kw OR (Maturity Onset Diabetes Mellitus):ab,ti,kw OR (MODY):ab,ti,kw OR (Diabetes Mellitus, Slow-Onset):ab,ti,kw OR (Diabetes Mellitus, Slow Onset):ab,ti,kw OR (Slow-Onset Diabetes Mellitus):ab,ti,kw OR (Type 2 Diabetes Mellitus):ab,ti,kw OR (Noninsulin-Dependent Diabetes Mellitus):ab,ti,kw OR (Noninsulin Dependent Diabetes Mellitus):ab,ti,kw OR (Maturity-Onset Diabetes):ab,ti,kw OR (Diabetes, Maturity-Onset):ab,ti,kw OR (Maturity Onset Diabetes):ab,ti,kw OR (Type 2 Diabetes):ab,ti,kw OR (Diabetes, Type 2):ab,ti,kw OR (Diabetes Mellitus, Adult-Onset):ab,ti,kw OR (Adult-Onset Diabetes Mellitus):ab,ti,kw OR (Diabetes Mellitus, Adult Onset):ab,ti,kw  #3 #1 or #2  #4 Erectile Dysfunction  #5 (Dysfunction, Erectile):ab,ti,kw OR (Male Impotence):ab,ti,kw OR (Impotence, Male):ab,ti,kw OR (Male Sexual Impotence):ab,ti,kw OR (Impotence, Male Sexual):ab,ti,kw OR (Sexual Impotence, Male):ab,ti,kw OR (Impotence):ab,ti,kw  #6 #4 or #5  #7 Phosphodiesterase 5 Inhibitors  #8 (Inhibitors, Phosphodiesterase 5):ab,ti,kw OR (PDE5 Inhibitor):ab,ti,kw OR (Inhibitor, PDE5):ab,ti,kw OR (Phosphodiesterase 5 Inhibitor):ab,ti,kw OR (5 Inhibitor, Phosphodiesterase):ab,ti,kw OR (Inhibitor, Phosphodiesterase 5):ab,ti,kw OR (PDE-5 Inhibitor):ab,ti,kw OR (Inhibitor, PDE-5):ab,ti,kw OR (PDE 5 Inhibitor):ab,ti,kw OR (PDE-5 Inhibitors):ab,ti,kw OR (Inhibitors, PDE-5):ab,ti,kw OR (PDE 5 Inhibitors):ab,ti,kw OR (PDE5 Inhibitors):ab,ti,kw OR (Inhibitors, PDE5):ab,ti,kw OR (Phosphodiesterase Type 5 Inhibitors):ab,ti,kw OR (Phosphodiesterase Type 5 Inhibitor):ab,ti,kw  #9 #7 or #8  #10 Medicine, Chinese Traditiona  #11 (Traditional Chinese Medicine):ab,ti,kw OR (Chung I Hsueh):ab,ti,kw OR (Hsueh, Chung I):ab,ti,kw OR (Traditional Medicine, Chinese):ab,ti,kw OR (Zhong Yi Xue):ab,ti,kw OR (Chinese Traditional Medicine):ab,ti,kw OR (Chinese Medicine, Traditional):ab,ti,kw OR (Traditional Tongue Diagnosis):ab,ti,kw OR (Tongue Diagnoses, Traditional):ab,ti,kw OR (Tongue Diagnosis, Traditional):ab,ti,kw OR (Traditional Tongue Diagnoses):ab,ti,kw OR (Traditional Tongue Assessment):ab,ti,kw OR (Tongue Assessment, Traditional):ab,ti,kw OR (Traditional Tongue Assessments):ab,ti,kw  #12 #10 or #11  #13 #3 and #6 and #9 and #12 | Cochrane Library | 1 |
| "TS= (Diabetes Mellitus, Type 2 or Diabetes Mellitus, Noninsulin-Dependent or Diabetes Mellitus, Ketosis-Resistant or Diabetes Mellitus, Ketosis Resistant or Ketosis-Resistant Diabetes Mellitus or Diabetes Mellitus, Non Insulin Dependent or Diabetes Mellitus, Non-Insulin-Dependent or Non-Insulin-Dependent Diabetes Mellitus or Diabetes Mellitus, Stable or Stable Diabetes Mellitus or Diabetes Mellitus, Type II or NIDDM or Diabetes Mellitus, Noninsulin Dependent or Diabetes Mellitus, Maturity-Onset or Diabetes Mellitus, Maturity Onset or Maturity-Onset Diabetes Mellitus or Maturity Onset Diabetes Mellitus or MODY or Diabetes Mellitus, Slow-Onset or Diabetes Mellitus, Slow Onset or Slow-Onset Diabetes Mellitus or Type 2 Diabetes Mellitus or Noninsulin-Dependent Diabetes Mellitus or Noninsulin Dependent Diabetes Mellitus or Maturity-Onset Diabetes or Diabetes, Maturity-Onset or Maturity Onset Diabetes or Type 2 Diabetes or Diabetes, Type 2 or Diabetes Mellitus, Adult-Onset or Adult-Onset Diabetes Mellitus or Diabetes Mellitus, Adult Onset) and Preprint Citation Index  "TS= (Erectile Dysfunction or Dysfunction, Erectile or Male Impotence or Impotence, Male or Male Sexual Impotence or Impotence, Male Sexual or Sexual Impotence, Male or Impotence) and Preprint Citation Index  "TS= (Phosphodiesterase 5 Inhibitors or Inhibitors, Phosphodiesterase 5 or PDE5 Inhibitor or Inhibitor, PDE5 or Phosphodiesterase 5 Inhibitor or 5 Inhibitor, Phosphodiesterase or Inhibitor, Phosphodiesterase 5 or PDE-5 Inhibitor or Inhibitor, PDE-5 or PDE 5 Inhibitor or PDE-5 Inhibitors or Inhibitors, PDE-5 or PDE 5 Inhibitors or PDE5 Inhibitors or Inhibitors, PDE5 or Phosphodiesterase Type 5 Inhibitors or Phosphodiesterase Type 5 Inhibitor) and Preprint Citation Index  "TS= (Medicine, Chinese Traditional or Traditional Chinese Medicine or Chung I Hsueh or Hsueh, Chung I or Traditional Medicine, Chinese or Zhong Yi Xue or Chinese Traditional Medicine or Chinese Medicine, Traditional or Traditional Tongue Diagnosis or Tongue Diagnoses, Traditional or Tongue Diagnosis, Traditional or Traditional Tongue Diagnoses or Traditional Tongue Assessment or Tongue Assessment, Traditional or Traditional Tongue Assessments) and Preprint Citation Index  "TS= (Randomized Controlled Trials as Topic or Clinical Trials, Randomized or Trials, Randomized Clinical or Controlled Clinical Trials, Randomized or Randomized Controlled Trial) and Preprint Citation Index  "#5 AND #4 AND #3 AND #2 AND #1 and Preprint Citation Index | Web of science | 0 |
| （主题：糖尿病）AND（篇关摘：勃起功能障碍 + 阳痿 + 阳萎(精确)）AND（篇关摘:中医 + 中药 + 中西医(精确)）AND（篇关摘：临床 + 随机对照(精确)） | CNKI | 76 |
| 主题:(糖尿病勃起功能障碍 OR 糖尿病阳痿 OR 糖尿病合并勃起功能障碍 OR 糖尿病性勃起功能障碍 OR 糖尿病阳痿) and 题名或关键词:(中医 OR 中药 OR 中西医) and 题名或关键词:(临床 OR 随机对照) | WANFANG | 24 |
| 主题:(糖尿病勃起功能障碍 OR 糖尿病阳痿 OR 糖尿病合并勃起功能障碍 OR 糖尿病性勃起功能障碍 OR 糖尿病性阳痿) and 篇关摘:(中医 OR 中药 OR 中西医) and 篇关摘：(临床研究 OR 随机对照研究 OR 随机对照试验 OR 临床试验) NOT 篇名：(研究进展OR 研究概况) | VIP | 119 |
